# Antibacterial evaluation of plants extracts against ampicillin-resistant *Escherichia coli* (*E. coli*) by microcalorimetry and principal component analysis

**DOI:** 10.1186/s13568-019-0829-y

**Published:** 2019-07-11

**Authors:** Zhuo Xu, Haotian Li, Xuhua Qin, Tao Wang, Junjie Hao, Jianwei Zhao, Jiabo Wang, Ruilin Wang, Dan Wang, Shizhang Wei, Huadan Cai, Yanling Zhao

**Affiliations:** 1 College of Pharmacy, Chengdu University of Traditional Chinese Medicine, Chengdu, 611137 China; 2 Department of Pharmacy, Fifth Medical Center, General Hospital of Chinese PLA, Beijing, 100039 China; 3 China Astronaut Research and Training Center, Beijing, 100094 China; 4 China Military Institute of Chinese Medicine, Fifth Medical Center, General Hospital of Chinese PLA, Beijing, 100039 China; 5 Integrative Medical Center, Fifth Medical Center, General Hospital of Chinese PLA, Beijing, 100039 China

**Keywords:** Microcalorimetry, *Dracontomelon dao*, Drug-resistant-*E. coli*, Principal component analysis

## Abstract

Antibiotics abuse has caused increased bacterial resistance, which severely limits the application of antibiotics to the treatment of bacterial infections. Therefore, it is urgent to develop new antibacterial drugs through other sources. *Dracontomelon dao* (Blanco) Merr. & Rolfe (Ren Mianzi in Chinese) is a traditional medicinal material derived from *Anacardiaceae* with a long history of treating various infectious diseases, such as decubitus and skin ulcers. Recent research has indicated that different extracts from the leaves of *D. dao*, especially the ethyl acetate (EtOAc) fraction containing flavonoids and phenolic acids, exhibit potent antibacterial activities. In this research, the combined anti-drug-resistant bacterial activities of these active ingredients were investigated. Six samples (S1–S6) were obtained from the EtOAc fraction of *D. dao* leaves. Microcalorimetric measurements and principal component analysis were performed on the in vitro samples. The results showed that all six samples had notable antibacterial activities. Specifically, sample S6 exhibited a prominent antibacterial effect, with an *IC*_50_ value of 84.3 μg mL^−1^, which was significantly lower than that of other samples. The relative contents of main flavonoids and phenolic acids in S6 sample were confirmed by UPLC/Q-TOF-MS. In conclusion, sample S6 from the EtOAc fraction of *D. dao* leaves could be used as a potential antimicrobial resource in the treatment of infectious diseases. This work provides an insight into the effect of traditional Chinese medicine on drug-resistant bacteria. Moreover, the purification and characterization of the chemical compounds from the sample S6 deserve further analysis.

## Introduction

From the discovery of penicillin in 1929 by Fleming to the end of the last century, great achievements have been made in developing anti-infective drugs, and bacterial infectious diseases have been effectively treated and controlled (Fleming 1980). However, the inappropriate use of antibiotics in clinical and non-clinical treatment has led to increased spread of bacterial resistance and damaged the human micro-ecological balance, often resulting in the failure of clinical antibacterial treatment. China is one of the countries where antibiotics abuse prevails. The number of nosocomial infections caused by drug-resistant bacteria accounts for about 30% of the total of hospitalized infections (Lima et al. 2013). Many pathogenic microorganisms have acquired multidrug resistance, including *Streptococcus pneumoniae* (*S. pneumoniae*), a pathogenic factor for a variety of common diseases, such as otitis media, pneumonia, and meningitis (Levy 2000). As a result of overuse, penicillin can no longer be used to effectively treat meningitis caused by *S. pneumoniae.* Due to the widespread abuse of antibiotics, the number of multidrug-resistant microorganisms (known as super bacteria) is increasing at a significant rate. These multidrug-resistant microorganisms will increase the rate of morbidity and mortality in related diseases. The National Center for Disease Control and Prevention (CDC) reported that in the United States, at least 2 million cases of severe infections are caused by one or more antimicrobial-resistant bacteria each year, among which 30,000 patients die from such infections (Thomas et al. 2019). The World Health Organization (WHO) reported in April 2014 that antibiotic-resistant bacteria are spreading over the globe and the world is entering a post-antibiotic era. Without any action, incurable infectious diseases and minor injuries could probably lead to death in the future, which may evolve into a global health crisis (Smith 2014).

Since the discovery of penicillin, the majority of antibiotic development has been focused on discovering new antibiotics from microbial sources or synthesizing new compounds using existing antibiotic scaffolds, which has however constrained the antibiotic development using other ways. While bacterial resistance to antibiotics has increased, the number of synthesized or discovered antibiotics has steadily reduced in the past ten years (Powers 2004). Therefore, researchers have to look for alternative therapies, including traditional plant drugs, phage therapy, and combination therapy. For example, pneumococcal conjugate vaccines can reduce the number of macrolide drugs used in hospitals for primary and second-line treatment, as well as decrease the morbidity of invasive pneumococcal diseases in children and adults (Lynch and Zhanel 2010). However, multi-drug resistance cloning appeared and threatened the success of vaccination. In the near future, Western medicine may consider phage therapy to be an effective substitute for antibiotics. Recent research has reported the success of phage therapy in treating antibiotic-resistant bacterial infections including methicillin-resistant *Staphylococcus aureus* (MRSA) and *Pseudomonas aeruginosa*, but further research is still required (Międzybrodzki et al. 2007; Jean-Marc et al. 2015).

Therefore, how to deal with the drug resistance of bacteria has become an urgent problem in the clinic. The development of low-toxicity and effective drug-resistant inhibitors is a potentially effective method to deal with bacterial resistance. In China, traditional treatments have relied on medicinal plants to treat bacterial infections for hundreds of years (Duraipandiyan et al. 2006). Approximately, 80% of the developing countries take the traditional medicines derived from medicinal plants as their primary health-care modality (Yadav and Agarwala 2011). Previous research has shown that Chinese herbal medicines contain broad-spectrum antimicrobial active ingredients, which are widely available, inexpensive, and have less toxic and side effects (Tomioka 2017; Li and Peng 2013). A WHO report mentioned that medicinal plants are one of the best potential sources of new drugs (Efferth 2017). There are numerous examples of compounds isolated from plants that have been verified to be effective as antimicrobial agents. Artemisinin, extracted from the plant *Artemisia annua* L., possesses antimalarial properties and has saved millions of lives globally (Burns et al. 2002). Resveratrol, which is found in grapes and Itadori plants (Paulo et al. 2010), exerts bacteriostatic effects on multiple Gram-positive and Gram-negative bacteria (Taylor et al. 2014; Schultes 1981). Consequently, searching for active ingredients from Chinese herbal medicines with activities against clinical multi-drug-resistant bacteria has become a research focus at the present time.

*Dracontomelon dao* (Ren Mianzi in Chinese) is a traditional medicinal material of *Anacardiaceae*. The leaves of *D. dao* have been widely used to treat various infectious diseases, such as decubitus and skin ulcers (Zhao et al. 2015). Moreover, the essential oil from the leaves of *D. dao* has been reported to have anti-tumor activity (Liu et al. 2014). Previous research by our team showed that different extracts from the leaves of *D. dao* exhibit different antibacterial activities, especially the ethyl acetate fraction containing flavonoids and phenolic acids (Wu et al. 2015; Li et al. 2017). However, previous research focused only on the evaluation of antimicrobial efficacy against model strains, without that against drug-resistant bacteria (Zhao et al. 2013).

Microcalorimetry refers to that under certain conditions, the thermal effect produced by the biochemical reaction induced by test substance is generated by static (constant temperature) continuous tracking measurement, with the characteristics of being fast, effective and sensitive. Moreover, the metabolic state could be monitored in an online, continuous and successive manner to determine the strength of drugs or chemicals. Finally, the microcalorimetry has the characteristic of high throughput with simple pretreatment to biosystem, including bacterium, cells, and organelles (Tafin et al. 2012). The admirable method has been widely used to evaluate the bioactive fraction of Chinese herbal medicines (CHMs) in recent years (Carvalho et al. 2013).

In this research, microcalorimetry and principal component analysis were performed to investigate the inhibitory effect of the samples obtained from the ethyl acetate part of *D. dao* leaves on the activity of drug-resistant *E. coli*. This research lays a foundation for the further development of antibacterial agents and provides an insight into the research of traditional Chinese medicine against drug-resistant bacteria.

## Materials and methods

### Samples, chemicals, and reagents

The leaves of *D. dao* (Batch No.: 20141013) were purchased from the CHMs market in Guangdong Province, China and authenticated by Professor Xiaohe Xiao (Chinese People’s Liberation Army (PLA) Institute of Chinese Material, Fifth Medical Center, General Hospital of Chinese PLA, Beijing, 100039, China). The leaves were dried in the shade and stored at room temperature.

Then, the leaves were crushed into powder and decocted for eight times with ultra-pure water by refluxing for 1.5 h. After the combined extract was filtered and evaporated, the water decoction was further extracted by EtOAc. Then the EtOAc fraction was eluted by 70% alcohol within column chromatography of polyamide and the samples (S1–S6) were detected by thin-layer chromatographic analysis and collected in succession by removing the solvent (Li et al. 2017). The six fractions were dried at low temperature and stored at 4 °C before the microcalorimetric experiment.

Polyamide for chromatography (60–100 mesh) was obtained from Mosu (Batch No.: 20160223, Shanghai Mosu Science Equipment Co., Ltd., Shanghai, China). 95% ethanol was from Lircon (Batch No.: 151111A, ShanDong LIRCON Medical Technology Co., Ltd., Shandong, China). Chromatographic grade methanol was from Sigma Chemicals (Batch No.: WXBC2019V, Sigma Scientific Co., L.L.C, USA).

The information of reference substances: Quercetin (the degree of purity ≥ 98%), Gallic acid (the degree of purity ≥ 98%), All reference substances were purchased from Chengdu Herbpurify bio-technology Co. Ltd., Chengdu, China.

### Bacterial strain and culture medium

*Escherichia coli* [BL21 (DE3) pLysS Chemically Competent Cell] was provided by TransGen Biotech, Beijing, China. According to the instruction manual, plasmid GFP (EX-EGFP-B01, Guangzhou FulenGen Co., Ltd, Guangdong, China) containing an additional ampicillin resistance gene was added to the 50 μL *E. coli* and ice bathed for 30 min. Next, it was water bathed at 42 °C for 45 s. Immediately after the tube was taken out of the water bath, it was placed on the ice again for 2 min. Shaking of the tube was prohibited in this process. Each tube was added with 500 μL sterile Luria–Bertani (LB) medium. Then, the bacteria were cultured at 37 °C and 200 RPM for 1 h for recovery. According to the experimental requirements, the transformed *E. coli* (GFP-*E. coli*) of different volumes were added to ampicillin LB agar medium. The plates were placed upside down at 37 °C overnight. The next day, a single colony was cultured in LB broth (Aobox Biotechnology, Beijing, China) at 37 °C. 100 μg/mL ampicillin (Amresco, Houston, Texas, USA) was used as antibiotics to select the bacteria containing GFP expression plasmid. Bacteria density was determined by the measurement of OD600, which was ca. 3.0 × 10^8^ colony-forming units (CFU/mL) for OD600 of 1.00.

The LB culture medium containing 10.0 g peptone, 5.0 g yeast extract and 5.0 g NaCl was dissolved in 1000 mL deionized water (pH of 7.0–7.2). Later, the culture medium was sterilized by autoclave at 121 °C and 0.1 MPa for 30 min and then stored in a refrigerator at 4 °C.

### Instruments and conditions

The thermal active monitor (TAM) air isothermal calorimeter (type 3114/3236, Thermometric AB, Sweden) was used to monitor the metabolic activity of the living cells of ampicillin-resistant *E. coli.* This microcalorimeter can records the thermal flux dQ/dt in microwatt range directly, also called heat-flow power *P*, which is different from classical calorimeters. The heat output Q value was obtained by integrating the metabolic power–time (*P–t*) curves using the TAM Assistant software (Thermometric AB, Stockholm, Sweden).

The bio-activity monitoring was conducted by a heat conduction calorimeter with eight channels for flow measurements under isothermal conditions. This microcalorimeter held a temperature at 37.00 °C ± 0.02 °C. The baseline stability was lower than 40 µW for 24 h. More details about the instrument can be found in the instruction and the report of Xie et al. (1988).

A UPLC/Q-TOF-MS liquid chromatography–mass spectrometry (type 6550, Ailent, USA) was used to analyze chemical composition information.

## Microcalorimetric measurement

### Samples preparation

Six samples were configured into 20 mg/mL solution with an appropriate amount of methanol. It was filtered through a micropore filter (pore size: 0.22 μm) for the measurement of trace calorimetry.

### Experimental procedure

This experiment was performed using an ampoule method and the microcalorimeter held a constant temperature of 37 °C (Zhao et al. 2014). All apparatus was cleaned and sterilized by autoclaving before use. Different concentrations of six samples from the leaves of *D. dao* contained in 9.0 mL LB culture medium was added into 20.0 mL sterilized glass ampoule. Each ampoule, except for the medium control group, should be guaranteed to contain the suspensions of ampicillin-resistant *E. coli* at the cell density of 1.0 × 10^6^ CFU/mL (Kong et al. 2011). Eventually, each ampoule was sealed up and put into the eight-channel calorimeter block. Before the thermodynamic curves were recorded, there was a 30 min pre-incubation. The *P*–*t* curves were recorded at an interval of 1 s until the recorder returned to the baseline (Kong et al. 2011). All data were collected continuously using the dedicated software package in a real-time manner.

### Principal component analysis (PCA)

The metabolic power–time (*P–t*) curves of ampicillin-resistant *E. coli* growth affected by sample S1–S6 were determined using the microcalorimetric method and then reconstructed by Origin 8.5 software (OriginLab Corp., Northampton, MA, USA) from which the antibacterial effects of the six samples on ampicillin-resistant *E. coli* can be observed intuitively. Then, metabolic parameters were obtained from the curves, which could be used to quantitatively assess the effects of the tested extracts. Because it is sometimes difficult to draw the final conclusion from too much information, representative parameters need to be captured.

Therefore, in order to provide a better solution for this problem, principal component analysis (PCA) is introduced, which is capable of reducing the dimensionality of redundant and noisy information from complex massive data and acquiring the important information that is compatible with the original data environment (David and Jacobs 2014). PCA can transform the original variables into two or three new orthogonal variables called principal components (PCs), which are uncorrelated to each other but contain nearly all of the original information. PC score plot indicates clear clustering of the concentration points from extracts, which is beneficial for elucidating the differences of antibacterial effects. From the loading plot, where each point corresponds to one of the quantitative parameters, the main parameters playing a crucial role in the effect evaluation can be identified and screened (Zhao et al. 2013). Here, PCA was carried out on mean-normalized data of the quantitative parameters from the power–time curve to find out the main parameter(s) by the SIMCA-P 13.0 software (Umetrics AB, Umea, Sweden) (Zhao et al. 2013).

## UPLC/Q-TOF-MS analysis

### Samples preparation

The standard solutions were prepared by accurately weighing 20.0 mg of Quercetin and Gallic acid added respectively into 10 mL of methanol to prepare the solutions with a concentration of 2 mg/mL, and then filtered through millipore filter with the pore size of 0.22 µm.

### UPLC conditions

A ZORBAX 300 SB-C18 (2.1 mm × 100 mm, 1.7 μm) was used for the chromatographic separation. The column temperature was maintained at 35 °C. The mobile phase consisted of acetonitrile solution (A) and 0.1% formic acid aqueous solution (B). The gradient elution progress was as follows: 5–50% A at 0–10 min, 50% A at 10–12 min, 50–100% A at 12–19 min, 100% A at 19–20 min. The flow rate was 0.25 mL/min. The autosampler temperature was maintained at 4 °C and the sample injection volume was 5 μL.

### Q-TOF-MS conditions

The temperature of the gasification chamber was 225 °C. The drying gas flow rate was 13 L/min. The sprayer pressure was 20 psi. The protective gas temperature was 275 °C. The protective gas velocity was 12 L/min. The capillary voltage was 3.5 kV. The typical nozzle voltage was 2.0 kV. The TOF debris voltage was 230 V. The OCT 1 RF Vpp voltage was 750 V. The mass number range of mass spectrometry was 50–1200 Da.

## Results

### Power–time curve of drug-resistant *E. coli* growth without intervention

**Fig. 1:**
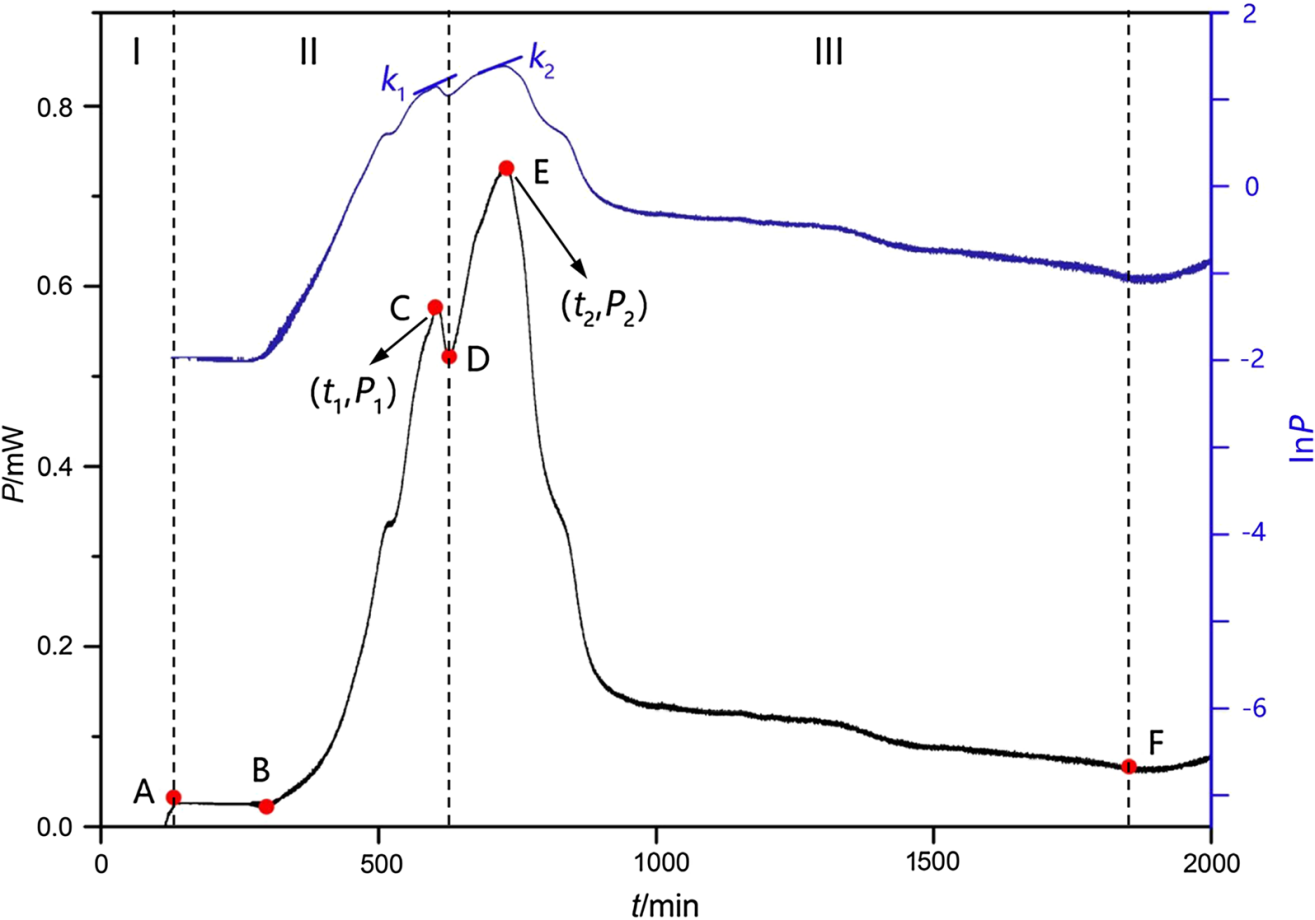
The metabolic curve of drug-resistant *E. coli* at 37 °C in the absence of any intervention: the *P–t* curve (the lower curve) and the corresponding Ln *P–t* curve (the upper curve). The straight line *k* is the linear fitting results of the corresponding phase

The metabolic process of drug-resistant *E. coli* growth was studied, and the metabolic thermogenic curve of drug-resistant *E. coli* at 37 °C without any exogenous intervention by taking advantage of the ampoule method has been shown in Fig. 1. The *P–t* curve exhibits the typical metabolic outline of drug-resistant *E. coli* growth culturing in the culture medium at 37 °C. Some important thermodynamic parameters that directly react with bacterial metabolism can be obtained. *P*_1_ and *t*_1_ are the heat output power and the corresponding appearance time of the first highest peak. *P*_2_ and *t*_2_ are the heat output power and the corresponding appearance time of the second highest peak. The Ln *P–t* curve shows the changing character of the growth and metabolism heat production. The straight-line *k* reflects the linear fitting results of the heat output power of the two highest peaks. As shown in Fig. 1, the growth and metabolism of the drug-resistant *E. coli* could be divided into three stages (stage I, II and III) and five phases, i.e., a lag phase (A–B), the first exponential growth phase (B–C), a stationary phase (C–D), the second exponential growth phase (D–E), and a decline phase (E–F). In stage I, while the ampoule bottles absorb heat and warm up, the instrument slowly reaches the equilibrium stage. In stage II, when the temperature reaches 37 °C, the bacteria begin to use oxygen in the limited air of ampoules for aerobic respiration growth and metabolism, and the first growth peak appears. In stage III, when the limited oxygen in the ampoule bottle is exhausted, the bacteria start to use the nutrients of LB medium for faster anaerobic fermentation metabolism, and the second growth peak appears. When the limited nutrients of the LB medium are depleted, the bacteria begin to decline until they die.

### Power–time curves and quantitative thermokinetic parameters for drug-resistant *E. coli* growth with six samples from the leaves of *D. dao*

**Fig. 2:**
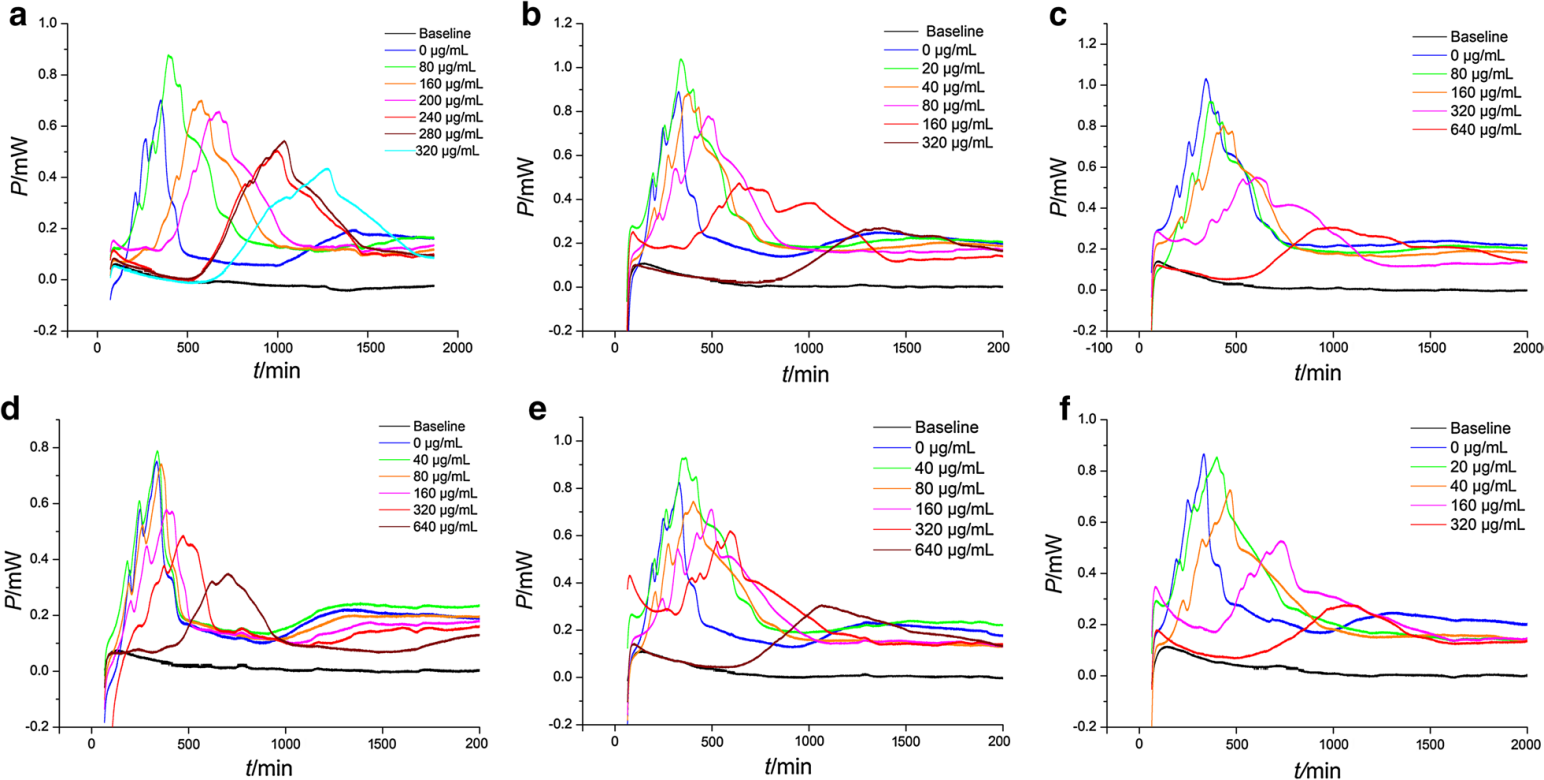
The power–time curves of drug-resistant *E. coli* growth with six samples from the leaves of *D. dao.* **a** Sample S1; **b** sample S2; **c** sample S3; **d** sample S4; **e** sample S5; **f** sample S6

The power–time curves of drug-resistant *E. coli* growth with six samples from the leaves of *D. dao* were measured and shown in Fig. 2. As could be suggested from the profiles of these curves, when the solutions of the six samples were added into the internal system of drug-resistant *E. coli* growth in the glass ampoules, the metabolism of the bacteria was influenced. Such influences could be intuitively seen from the heights and occurrence frequency of the peaks in Fig. 2, such as the peak reducing, the time of peak postponing, the slope of curves diminishing and the area of curves lowering. The results in Fig. 2 indicate that within a certain concentration range, the samples showed different effects on drug-resistant growth. But, these curves are similar and the five phases still exist.

From the metabolic power–time curves of *E. coli* growth (Fig. 2), in the exponential growth phase, the heat-output power *P* of bacterium growth obeys the following kinetic equation:

1 $$P_{t} = P_{0} \exp \left( {kt} \right)\quad {\text{or}}\quad {\text{In}}P_{t} = \ln P_{0} + kt$$

where *P*_0_ represents the heat-output power at time *t* = 0, and *P*_*t*_ represents the power at time *t*. Therefore, using the data of ln *P*_*t*_ and *t* obtained from the curves to fit a linear Eq. (1), the growth rate constants (*k*_1_ and *k*_2_) in the first and second exponential growth phase were calculated for quantitatively describing the growth status of the bacteria. In addition, other thermokinetic parameters, such as the heat output *Q*_1_ for stage II and the *Q*_2_ stage for III could be obtained. Also, the total heat output *Qt* can be calculated, as listed in Table 1.

**Table 1:** Thermokinetic parameters of ampicillin-resistant *E. coli* growth in the presence of six samples from the leaves of *Dracontomelon dao*

Extracts	c/μg mL^−1^	*t*_1_/min	*P*_1_/mW	*Q*_*1*_/J	*k*_1_	*t*_2_/min	*P*_2_	*Q*_*2*_ */J*	*k*_2_	Q_t/_J	I/%
S1	0	267.2	0.5511	2.6079	0.01126	350.1	0.7023	27.1152	0.00929	29.7231	0.0
	80	306.6	0.5447	3.7603	0.00995	393.9	0.8785	17.4783	0.00896	21.2386	3.5
	160	439.5	0.4069	3.4218	0.00565	573.9	0.7009	17.1311	0.00527	20.5529	43.2
	200	532.6	0.4276	4.7574	0.00433	672.6	0.6575	16.7857	0.00407	21.5431	56.2
	240	819.8	0.376	3.1187	0.00304	989.5	0.505	13.9117	0.00282	17.0304	69.6
	280	845.6	0.3885	3.4423	0.00295	1035.1	0.5434	15.3056	0.00274	18.7479	70.5
	320	1047.0	0.3245	4.4208	0.00210	1274.6	0.4362	12.3450	0.00196	16.7658	78.9
S2	0	246.8	0.7255	3.6592	0.01331	327.4	0.8907	40.9180	0.01066	44.5771	0.0
	20	256.5	0.739	5.2648	0.01308	338.3	1.0397	19.5135	0.01093	24.7783	0.0
	40	274.7	0.6024	3.9038	0.01047	377.9	0.8822	34.8650	0.00862	38.7688	19.2
	80	310.1	0.5412	4.7950	0.00922	480.0	0.7802	21.7137	0.00672	26.5086	36.9
	160	535.3	0.371	6.4258	0.00458	641.9	0.4759	17.5933	0.00421	24.0191	60.5
	320	1281.3	0.2638	6.0494	0.00156	1376.7	0.2717	15.2971	0.00147	21.3465	86.2
S3	0	255.5	0.7245	5.4127	0.01285	344.7	1.0325	76.7405	0.01055	82.1532	0.0
	80	256.5	0.5023	2.4554	0.01157	362.4	0.921	70.7951	0.00986	73.2505	6.5
	160	305.9	0.5422	5.0593	0.00905	433.2	0.8036	73.0303	0.00730	78.0896	30.8
	320	374.2	0.3447	5.1102	0.00647	605.5	0.5525	34.5903	0.00476	39.7004	54.9
	640	94.5	0.1238	1.9171	0.01432	978.1	0.3072	37.6609	0.00231	39.5779	78.1
S4	0	251.1	0.5814	2.5884	0.01220	327.7	0.7375	52.1562	0.01007	54.7446	0.0
	40	245.5	0.6108	3.6068	0.01289	338.7	0.7896	57.5782	0.01010	61.1850	0.0
	80	262.1	0.5237	3.0778	0.01043	357.3	0.7423	58.3756	0.00863	61.4534	14.3
	160	282.4	0.4486	2.8312	0.00946	386.6	0.5798	56.9032	0.00758	59.7344	24.8
	320	373.1	0.3794	3.0034	0.00663	471.7	0.4864	59.7257	0.00577	62.7290	42.7
	640	619.0	0.323	4.1183	0.00355	705.8	0.3506	7.6208	0.00323	11.7390	67.9
S5	0	232.7	0.5773	2.6695	0.01313	329.4	0.8252	56.5456	0.01036	59.2150	0.0
	40	263.6	0.7121	5.2766	0.01259	363.8	0.9302	79.0143	0.00986	84.2910	4.9
	80	274.2	0.5664	3.5835	0.01026	402.7	0.7452	45.8086	0.00767	49.3921	26.0
	160	421.8	0.6124	7.4357	0.00707	495.0	0.7121	61.1381	0.00633	68.5738	38.9
	320	529.5	0.5756	6.0004	0.00546	591.1	0.6197	57.5773	0.00501	63.5777	51.6
	640	845.7	0.1652	3.0030	0.00180	1058.8	0.3052	33.0030	0.00202	36.0060	80.5
S6	0	247.9	0.6882	4.4994	0.01303	332.0	0.8665	42.6671	0.01043	47.1665	0.0
	20	280.6	0.6708	5.5756	0.01161	398.0	0.8547	35.9981	0.00879	41.5737	15.6
	40	388.6	0.5994	5.9808	0.00739	467.6	0.7267	34.1850	0.00655	40.1657	37.2
	160	657.2	0.4864	9.9455	0.00419	730.0	0.5284	26.5580	0.00389	36.5035	62.7
	320	968.0	0.2628	6.6450	0.00218	1059.8	0.2786	24.9284	0.00204	31.5733	80.4

As shown in Fig. 2, it could be found that the changes of bio-active fingerprints of growth and metabolism of drug-resistant *E. coli* in the presence of different samples from the leaves of *D. dao* visually and qualitatively. These changes can be further reflected in Table 1 objectively and quantitatively. However, it is difficult to accurately evaluate the antibacterial effects of the different extracts due to the overlapping of information of the redundant parameters, and the disordered fluctuation. So, it is necessary to choose the main parameters to evaluate the effects in a fast and accurate way. Therefore, principal component analysis (PCA) is adopted in this research. The drug-resistant *E. coli* growth in the six samples could be quantitatively reflected from these changes of nine important thermokinetic parameters in Table 1.

### PCA on the thermokinetic parameters

**Fig. 3:**
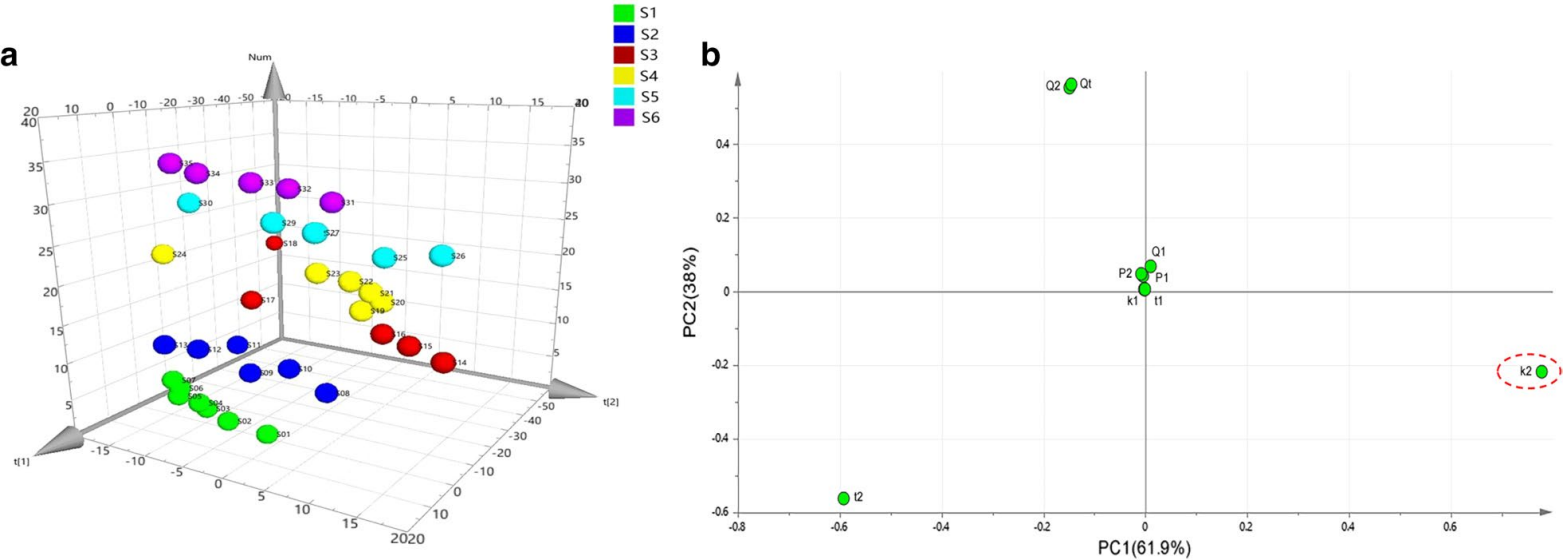
Results of PCA on the quantitative parameters from the power–time curves of drug-resistant *E. coli* growth affected by six samples from the leaves of *D. dao.*** a** 3D PCA score plot counted from the supervised PLS-DA where each point corresponds to a single concentration level of the samples, showing clear purple sphere clustering of the concentration points from the S6 sample. **b** Loadings plot indicated the contribution of the original variables (parameters) to the strength of the first two principal components PC1 and PC2, so that the main parameter(s) of the effect evaluation can be found and marked with a circle

PCA can transform the multidimensional variables into two or three variables called PCs, which contain nearly all of the original information. In order to further investigate the tendency and internal change rule of different polarities on the inhibitory effects, the values of nine parameters in Table 1 were processed by PCA and the results have been illustrated in Fig. 3. The parameters in the original nine-dimensional space were projected to the new three-dimensional space with the first two PCs (PC1 and PC2). The variance contribution rate of the first principal component PC1 is 61.9%, the variance contribution rate of the second principal component PC2 is 38%, accounting for 99.9% of the information of the original data set cumulatively. The 3D PCA score plot in Fig. 3a, each point corresponded to a single concentration level of the samples from the leaves of *D. dao*, demonstrating clear gathering of the concentration points from the samples. These above-mentioned findings further demonstrated different antibacterial effects of the six samples.

According to the corresponding loadings plot in Fig. 3b indicated that the parameter *k*_2_ was the farthest away from the other eight parameters and contributed more for PC1 and PC2, which might be the main quantitative parameter that played a crucial role in evaluating and comparing the bacteriostatic effects of the six samples and marked with a circle.

### Growth percent inhibition (I) and half inhibitory concentration (*IC*_50_)

To quantitatively evaluate the effect when drug-resistant *E. coli* was treated with different concentrations of fractions, another necessary parameter, based on the main parameter *k*_2,_ the growth percent inhibition (*I*%) was calculated, which is defined as.

2 $$I = \, \left[(k_{0} - k_{c} )/k_{0} \right] \, \times 100 \, \%$$

where *k*_0_ is the growth rate constant of the control (without any sample), and *k*_*c*_ is the growth rate constant of the second exponential growth phase. The higher the value of *I*, the stronger the inhibitory effect of the samples on drug-resistant *E. coli*. The results were presented in Table 1.

Furthermore, based on the values of *I*, the probit regression with IBM SPSS 22.0 software (Chicago, United States) was conducted to calculate the half-inhibitory concentration (*IC*_50_) of the samples on drug-resistant *E. coli*, which was the concentration that caused 50% inhibition (*I* = 50%) on drug-resistant *E. coli* growth and have been listed in Table 2. *IC*_50_ was one of the most important indicators for evaluating the antibacterial effects of many materials and it represented the sensitivity of bacteria to these materials. So Table 2 can directly show the relationships between the antibacterial effect, the *IC*_50_ of 190.4 μg mL^−1^ for S1, the *IC*_50_ of 114.6 μg mL^−1^ for S2, the *IC*_50_ of 289.2 μg mL^−1^ for S3, the *IC*_50_ of 372.0 μg mL^−1^ for S4, the *IC*_50_ of 242.4 μg mL^−1^ for S5, the *IC*_50_ of 84.3 μg mL^−1^ for S6 on *E. coli* were obtained, which meant that 190.4 μg mL^−1^ of S1, 114.6 μg mL^−1^ of S2, 289.2 μg mL^−1^ of S3, 372.0 μg mL^−1^ of S4, 242.4 μg mL^−1^ of S5, 84.3 μg mL^−1^ of S6 could cause a 50% decrease of the drug-resistant *E. coli* growth rate constant.

**Table 2:** Half inhibition ratios of S1–S6 (95% confidence limits) and the correlation coefficient R

Extracts	IC50/μg mL^−1^	Lower limit/μg mL^−1^	Uper limit/μg mL^−1^	R
S1	190.4	177.2	203.1	0.974
S2	114.6	79.4	174.6	1.906
S3	289.2	252.4	334.3	0.963
S4	372.0	255.5	674.3	0.954
S5	242.4	157.1	426.8	0.934
S6	84.3	67.3	105.9	0.944

These results have obviously suggested that all the six samples had anti-drug-resistant *E. coli* activities which following a sequence of S6 > S2 > S1 > S5 > S3 > S4, which indicated that the sixth fraction of the EtOAc extracts of the leaves of *D. dao* expressed the strongest antibacterial effect. The value of *IC*_50_ for sample S6 was 84.3 μg mL^−1^. The mechanism of the anti-bacterial effects of the leaves of *D. dao* on drug-resistant bacteria was in process in our work.

### UPLC/Q-TOF-MS analysis

Importing mass spectrometry data into Agilent Mass Hunter Qualitative Analysis B.06.00 software, after calibration and standardization, comparing with TCM database and identified by reference substances, the results showed that quercetin and gallic acid were main compounds in S6 sample. The relative content of the compounds can be characterized by the response strength of the ions. The response strength of Quercetin and Gallic acid signal ions were 236608263.14 and 250475383.14 respectively. The relative content of main flavonoids and phenolic acids can be characterized by this method.

## Discussion

Many evidences have shown that the extracts from medicinal plants are effective as antimicrobial agents. Moreover, no reports have claimed to have observed bacteria developing resistance to plant-based antimicrobials (PBAs) (Cheesman et al. 2017). *D. dao*, a traditional Chinese medicine, has been used for the treatment of various skin infectious diseases over 1000 of years. Previous reports have demonstrated that the leaves of *D. dao* present favorable antibacterial activity against *Escherichia coli*, *Pseudomonas aeruginosa*, *Staphylococcus aureus*, and *Bacillus subtitles*. However, the above research focused only on the evaluation of antimicrobial efficacy against model strains.

In this research, an investigation was performed on the anti-drug-resistant *E. coli* activities of six samples (S1–S6) from the purified active fraction in the leaves of *D. dao* by microcalorimetry. Using this microcalorimetric method, the whole metabolism of the microbes can be examined automatically and continuously, and some critical information including the real-time metabolic power–time curve together with a variety of quantitative thermokinetic parameters of drug-resistant *E. coli* growth in the presence of the test fractions was obtained. Traditional microbiological methods, including agar cup method and continuous dilution method, could not do this in the evaluation of the antimicrobial activity. Combined with PCA, a multivariate analysis method, widely used in laboratory, clinical and epidemiological research, the antibacterial effects of the six samples on *E. coli* were quickly and systematically evaluated. This may help to reduce the number of conflicting reports of fractions in TCM antibacterial activity in the future. It was found that all six samples had different antibacterial activities. Specifically, sample S6 had a very prominent antibacterial activity, with the value of *IC*_50_ being 84.3 μg mL^−1^. The chemical compounds isolated from sample S6 deserve further analysis. The previous research has shown that flavonoids and phenolic acids are abundant in the EtOAc extracts of *D. dao* leaves so they might play crucial roles in the anti-resistant bacteria effect. We used UPLC/Q-TOF-MS to make a preliminary exploration of the chemical composition of S6 samples. Its main components were quercetin and gallic acid. Their anti-bacterial activities have been reported by cumulative evidence. For example, flavonoids are against both Gram-positive and Gram-negative bacteria in the ethanolic extracts from the leaves of *Combretum album* (Sunanda et al. 2018), a phenolic acid isolated from the flowers of *Trollius chinensis* has anti-inflammatory and antibacterial effects (Li et al. 2014). Moreover, the monomer composition of flavonoids and phenolic acid still have antibacterial activity. Experimental research has found that naringenin has good antibacterial activity against *E. coli*, *S. aureus* and *B. subtilis* (Zhang et al. 2013). Rita et al. (2016) applied the disk diffusion method to test the antimicrobial activity of gallic acid on *Staphylococcus aureus*, *Escherichia coli*, *Salmonella enteritidis*, *Salmonella typhimurium*, *Bacilluscereus* and *Candida albicans*, and the result showed that galic acid had antimicrobial activity against all of these bacteria. The mechanism of the anti-bacterial effects of the leaves of *D. dao* on drug-resistant bacteria was in process in our work.

Based on our experimental results and the broad-spectrum antimicrobial activities of flavonoids and phenolic acids, it is suggested to further isolate and purify flavonoids and phenolic acid monomers from the sample S6, to explore their antimicrobial activities against other drug-resistant strains. Once the antibacterial monomer is found, it is possible to produce a better antibacterial compound by optimizing its chemical structure. In later research, we will divide it into two directions. On the one hand, we will extract the monomers and analyze their antimicrobial activity in depth. On the other hand, because the existing studies are all in vitro experiments, we consider carrying out in vivo animal experiments to investigate their antimicrobial activity in an all-around way. In summary, this study introduced the useful ideas and tools of combining microcalorimetry with PCA for evaluating the antimicrobial effect of traditional Chinese medicine and provided help for better research and development of new antimicrobial drugs in the future.

## Data Availability

Not applicable.
